# Effect of Size and Surface Charge of Gold Nanoparticles on their Skin Permeability: A Molecular Dynamics Study

**DOI:** 10.1038/srep45292

**Published:** 2017-03-28

**Authors:** Rakesh Gupta, Beena Rai

**Affiliations:** 1 Physical Science Research Area, TCS Research, Tata Research Development and Design Centre, Tata Consultancy Services, 54B, Hadapsar Industrial Estate, Pune – 411013, India

## Abstract

Molecular level understanding of permeation of nanoparticles through human skin establishes the basis for development of novel transdermal drug delivery systems and design and formulation of cosmetics. Recent experiments suggest that surface coated nano-sized gold nanoparticles (AuNPs) can penetrate the rat and human skin. However, the mechanisms by which these AuNPs penetrate are not well understood. In this study, we have carried out coarse grained molecular dynamics simulations to explore the permeation of dodecanethiol coated neutral hydrophobic AuNPs of different sizes (2–5 nm) and surface charges (cationic and anionic) through the model skin lipid membrane. The results indicate that the neutral hydrophobic AuNPs disrupted the bilayer and entered in it with in ~200 ns, while charged AuNPs were adsorbed on the bilayer headgroup. The permeation free energy calculation revealed that at the head group of the bilayer, a very small barrier existed for neutral hydrophobic AuNP while a free energy minimum was observed for charged AuNPs. The permeability was maximum for neutral 2 nm gold nanoparticle (AuNP) and minimum for 3 nm cationic AuNP. The obtained results are aligned with recent experimental findings. This study would be helpful in designing customized nanoparticles for cosmetic and transdermal drug delivery application.

Modeling of human or animal skin has been an interesting area of research for pharmaceutical and cosmetics industries. The fate of the therapeutic agents is decided by the permeation mechanism of these agents through skin for novel transdermal drug delivery system. In addition, minimization of permeation of active agents (nanoparticles, molecules and so on) is important for both the cosmetic industry and health and safety regulations in an industrial environment. The human skin is one of the largest organs of the body and provides a convenient route of administration of drugs because of its high surface area and the drug can also be self-administered^1^. However, due to its morphology, skin does not allow high molecular weight drugs or proteins and hydrophilic drugs to permeate inside itself 2. *Stratum Corneum* (SC), the upper most layer of epidermis, is mainly responsible for these barrier properties^3^. The molecules are transported across SC primarily by passive diffusion^4^. SC is highly selective in nature and allows only relatively small lipophilic compounds to diffuse into the inner layers of skin. Therefore, to deliver the therapeutic agents through skin, the SC barrier has to be breach. In recent years, extensive experiments have been carried out to breach the skin barrier^5^,^6^. On the basis of applications, these experiments have been classified in two categories, namely, passive and active methods. Chemical penetration enhancers and liposomes (passive method) interact with the skin constituents and change the morphology of skin at molecular scale^5^. Active methods such as electroporation, iontophoresis, sonophoresis and thermophoresis use external energy source (electric current, ionic flux and so on) to create the temporary nano-pores in the SC that lead to the permeation of molecules through skin^6^. The active methods although faster than passive methods, but induce irritation and damage to the skin barrier function up to undesired extent. Some of the permeation enhances such as ethanol^7^ and di-methylsufroxide (DMSO)^8^ perturb the skin morphology significantly. In order to overcome some of the negative effects associated with permeation enhancers such as ethanol and DMSO, researchers have shown considerable interest in nanoparticles^9^, such as gold nanoparticles, due to their easy surface modification by either chemical compound or bioactive molecules^10^,^11^.

Several experimental studies of permeation of AuNPs through human and rat skin have been reported^12^,^13^,^14^,^15^,^16^,^17^,^18^,^19^ Dean *et al*.^12^ showed that the deoxyribonucleic acid coated AuNPs enhanced the permeation through outer layer of the skin to Langerhans cells. Chithrani *et al*.^13^,^14^ studied the effect of size, shape, and chemical surface of AuNPs on their cellular uptake. Nativo *et al*.^15^ demonstrated that the well-established endosomal route of cellular uptake can be bypassed to a significant extent by controlling the uptake mechanism through the delivery of the nanoparticles by liposomes or by surface modification of the nanoparticles with cell penetrating peptides. Sonavane *et al*.^16^ studied the *in vitro* permeation of AuNPs of different sizes through rat skin and showed that the smaller AuNPs penetrated in the deeper layer of the skin. Huang *et al*.^17^ reported co-delivery of proteins using 5 nm AuNP through rat skin. Labouta *et al*.^18^ studied the penetration of thiol coated 6 nm AuNP through human skin using multiphoton imaging pixel analysis. The same author also reported the effect of surface modifications, size, vehicles, and concentrations of AuNPs over human skin permeation^19^. Larese *et al*.^20^ performed permeation experiments on intact and damaged human skin and reported that the AuNPs penetrated deeper in the layer and flux of AuNPs for damaged skin was an order of higher than that of intact skin. Fernandes *et al*.^21^ studied the interactions between the skin and colloidal AuNPs of different physicochemical characteristics by systematically varying the charge, shape, and functionality of gold nanoparticles. The AuNPs functionalized with cell penetrating peptides TAT and R7 were found larger in quantities than polyethylene glycol functionalized AuNPs inside the skin^21^.

Above mentioned studies show that bare AuNPs and surface modified nanoparticles can penetrate into the viable tissues. In contrast, Liu *et al*.^22^ showed that viable human skin resists permeation of small nanoparticles in a size range that has been reported to penetrate deeply in other skin models. Despite of having numerous experimental studies, there is no clear understanding among researchers about the penetration of surface modified or coated nanoparticles through SC or into the viable tissue.

Molecular simulations provide a convenient way to understand mentioned permeation processes and can yield important physical insights at molecular levels that could not be obtained from experiments because of associated time and length scale^23^. In terms of modeling, few studies have been reported on the interactions of AuNPs with model cell lipid membranes. A Kyrychenko *et al*.^24^ synthesized thiol coated colloidal AuNP of 3.75 ± 0.06 nm and based on their experiments, a new Coarse Grained (CG) model of AuNP was developed. JQ Lin *et al*.^25^,^26^,^27^ developed a CG model for AuNPs and verified against experimental data. Using these force fields, it was established that both the level of penetration and membrane disruption increased with the charge density of the AuNP^25^. It was also shown that AuNPs functionalized with cationic ligands penetrated the negative bilayer membranes and generated significant disruptions in bilayers^26^. Same research group investigated the dynamics of 2.2 nm monolayer protected AuNPs in solvents. The effects of ligand length, ligand terminal chemistry, solvents, and temperature were also studied^27^. Simonelli *et al*.^28^ studied the kinetics and the thermodynamics of the interaction between the anionic ligand protected AuNPs and the model lipid membranes using CG molecular dynamics (MD) simulations. The AuNPs and membrane interaction was shown to be a three-step process: electrostatics driven adhesion to the membrane surface, hydrophobic contact and final embedding in the membrane core via anchoring of the charged ligands to both membrane leaflets^28^. We have recently reported on the permeation of different sized bare AuNPs with skin^29^. We reported that a tradeoff between thermodynamics and kinetics of nanoparticle permeation, lead to the higher permeation of small sized bare AuNPs through human skin lipid model^29^.

In this study, we reported the interaction between the various AuNPs and skin lipids and permeation of surface coated neutral hydrophobic, anionic and cationic gold nanoparticles through model skin membrane. The constrained and unconstrained CG MD simulations of skin lipid membrane with neutral and charged gold nanoparticles. The neutral gold nanoparticles (2 nm–5 nm) were coated with dodecanethiol and were in hydrophobic in nature. The effect of surface charge was studied for the 3 nm AuNP only. Several constrained CGMD simulations were performed to calculate the potential of mean force, diffusion coefficient and permeability of different size and charge containing AuNPs through skin lipid bilayer.

## System, Models and Methods

The SC is made of corneocytes (brick) and lipid matrix (mortar)^3^. The lipid matrix is considered to be a key determinant for the barrier functions because the corneocytes are almost impermeable. The lipid matrix is mainly composed of heterogeneous mixture of long chain ceramides (CER), cholesterol (CHOL) and free fatty acids (FFA)^30^,^31^. Based on the structure of head group and attached fatty acid chain length, there are more than 300 different types of CER present in the SC. Simulation of skin lipid model, comprising of all kinds of CER, is beyond the current computational capability. In order to simulate a realistic SC layer^30^, we have chosen the most abundant ceramide, CER-NS (24:0) and free fatty acid, FFA (24:0). As in this study, only single kind of ceramide and fatty acid are used, henceforth CER-NS 24:0 and FFA 24:0 will be denoted by CER and FFA respectively throughout the paper unless it stated otherwise.

**Figure 1:**
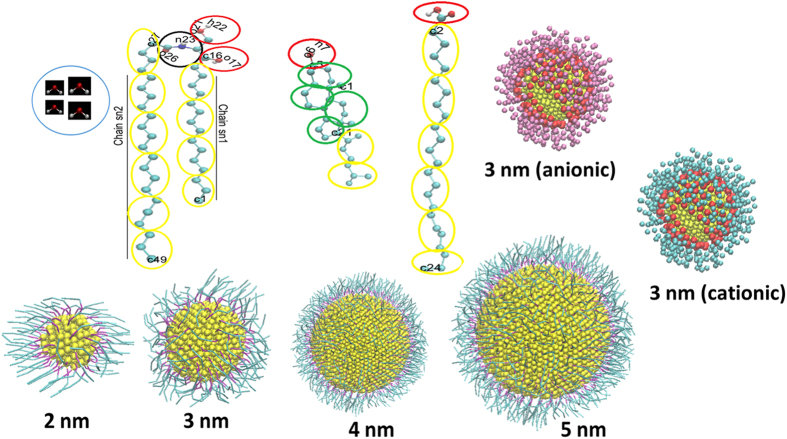
Coarse grained structure of skin lipid molecules, water and different AuNPs. CG molecular structures of individual components CER, CHOL, FFA of skin lipid matrix, water and thiol coated gold nanoparticles of different sizes used in the simulation. Images/snapshots were created using the VMD software^59^. The sn1 and sn2 represents the chains of CER. Images are not drawn to scale.

In order to model the heterogeneous mixture of CER, FFA, CHOL and AuNPs at the realistic time and length scale, the CG models were used. The CG model of skin lipid bilayer was obtained from the earlier developed united atom model^32^. The CG models were based on the MARTINI force field^33^,^34^. The CG parameters for CER were taken from the recent work of Sovova *et al*.^35^. The CG parameters for CHOL and FFA were taken from our earlier work^29^. The CG parameters of thiol coated AuNPs were taken from previous simulation studies^25^,^26^,^27^,^29^. The simulations were carried out in NVT and NPT ensemble using the GROMACS MD package^36^,^37^,^38^. The temperature was controlled at a skin temperature of ~ 310 K, using the Berendsen (equilibration run) and Nose-Hover (production run) thermostat with a time constant of 2 ps. Pressure was controlled by Berendsen (equilibration run) and Parrinello-Rahman (production run) barostat with a time constant of 6 and 12 ps respectively and compressibility of 4.0 × 10^−5^ bar^−1^ with semi-isotropic coupling. The pressure was controlled in *XY* and *Z* direction independently to obtain the tensionless bilayer. The LJ potentials were smoothly shifted to zero between a distance *r*_*shift*_ = 0.9 nm and the cutoff distance of 1.2 nm. The pair list was updated at every 20 steps. The configuration was sampled at every 50 ps in production run. The CG model of CER, CHOL, FFA, neutral hydrophobic and charged AuNPs used in this study are shown in Fig. 1. The CG structure of skin lipid bilayer (equilibrated for 3 μs) and bare AuNPs were taken from our earlier work^29^. CG dodecanethiol chains were attached to the surface atoms of the bare AuNPs using an in-house python script. For modeling the charged AuNPs, the terminal beads of thiol chain of coated AuNP (3 nm) were assigned a charge of +1 or −1 for cationic and anionic AuNP, respectively. The number density of surface charges was calculated by


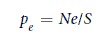


where N is the number of surface beads, e is the reduced charge of one surface bead, and S is the surface area of NPs. The charge density on both cationic and anionic gold nanoparticle was ~+5 e/nm^2^ and ~−5 e/nm^2^ respectively. Adequate numbers of counter ions were added to the system to maintain the charge neutrality. To allow complete inclusion of AuNPs and to avoid artifacts associated with the system size and AuNP-AuNP interactions over periodic boundaries, the water content (Table 1) was increased on the each side of the bilayer (the one obtained from our earlier after 3 μs run)^29^. The AuNPs were inserted in the upper part of the water layer at the distance of ~7–8 nm from the center of the lipid bilayer. The overlapped water molecules were removed and systems were energy minimized. The systems were further equilibrated for 200 ns each in NVT and NPT conditions by keeping lipids and AuNPs fixed to ensure the proper solvation. Properly solvated systems were then run for 3 μs in NPT ensemble.

The passive permeability of molecules through membrane is generally calculated using homogenous solubility diffusion model. The solute first dissolves into the membrane, then diffuses through the membrane interior, and finally dissolves again in the outer surrounding medium. However; the lipid bilayer’s interior is heterogeneous in nature. MD simulation provides an attractive way to calculate free energy of permeation d*G(z)* and local diffusion coefficient *D(z)* along the bilayer normal *z*. The non-homogeneous solubility diffusion model was used to compute the diffusivity and free energy^39^,^40^. In this model, the permeability for a symmetric bilayer system, which has the normal in *z* direction, is given by


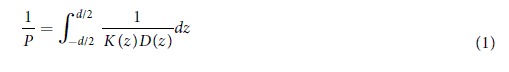


where, *P* is the permeability, *d* is thickness of the bilayer. *K(z)* and *D(z)* is partition function and diffusion coefficient at a given *z* position from the center of bilayer that are calculated using following equations


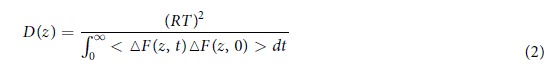



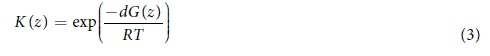



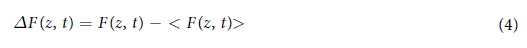



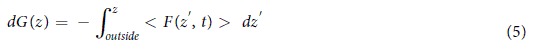


where, *R* is gas constant, *T* is temperature, *F(z, t)* is constrained force on solute at a given z and *dG* is free energy. In constrained simulations, the reaction coordinate of the system was chosen to be membrane normal z, where z = 0 nm corresponds to the center of mass (COM) of the bilayer. In this study, the separation distance z corresponds to distance between the ***z*** coordinates of COM of Au particle and COM of membrane. More details on calculation of average force, free energy and diffusion are provided in supporting information (Fig. S4). For each system, the AuNP was placed manually at z distance of ~7–8 nm from the COM of the bilayer according to the size of AuNP. Overlapped water molecules with AuNP were removed and system was then energy minimized. Each system was then equilibrated in NPT ensemble for 1 μs by keeping AuNP fixed at their positions. These equilibrated structures were further used for the preparation of the initial configuration for constrained simulations. AuNP was pulled slowly at the rate of 0.002 nm/ps towards the center of the bilayer. The time step for this run was kept at 20 fs. As the z distance between the COM of the lipid and AuNP changed by 0.2 nm, the configuration was stored. Using this procedure 40 windows were generated. These windows spanned the whole space from the upper bulk water to the middle of the bilayer. The stored equidistance configurations were further run for 200 ns out of which first 120 ns simulation was discarded as an equilibration run. The distance between the COM of AuNP and COM of bilayer was constrained in z direction while AuNP was free to move in lateral direction. The configuration was stored at every 50 ps and constrained force was stored at every 100 fs. Last 80 ns runs of each simulation were used to calculate the thermodynamic and transport properties. As the bilayer was symmetric, the results from one leaflet of bilayer were replicated for the other leaflet.

## Results and Discussion

### Effect of Size: Unconstrained Simulation

**Figure 2:**
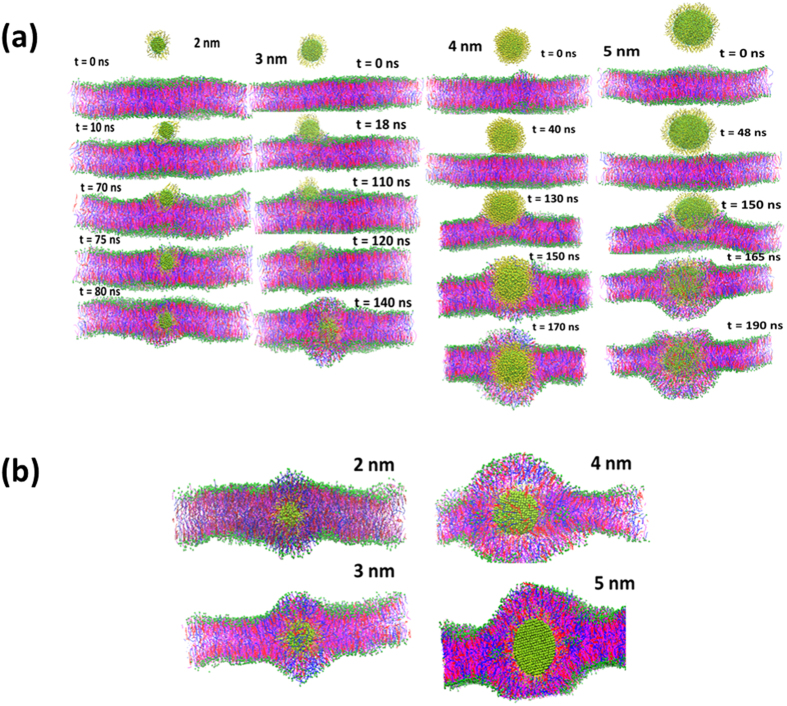
Results of interaction between different sized neutral hydrophobic AuNPs and skin lipid bilayer in unconstrained simulation. (**a**) Time sequence of structural changes induced by the thiol coated AuNPs (neutral hydrophobic) of size of 2 nm, 3 nm, 4 nm and 5 nm into skin lipid bilayer. (**b**) Snapshot of thiol coated AuNPs (neutral hydrophobic) of size of 2 nm, 3 nm, 4 nm, 5 nm bilayer in the end of unconstrained 3 μs MD run. The head groups of lipids (CER, CHOL and FFA), CER tail, CHOL aromatic rings, FFA tail, thiol chain and AuNP are shown in green, magenta, red, blue, yellow and dark green color respectively. Water molecules were removed for the purpose of clarity. Images/snapshots were created using the VMD software^59^.

The AuNPs were initially placed at the distance of almost ~7–8 nm above the bilayer center (z = 0) in the upper water layer. The results of interaction between the neutral hydrophobic AuNPs (2–5 nm) and skin lipid bilayer are shown in Fig. 2a. Each AuNP first translated within the water layer for first few nanoseconds and then started to move towards the bilayer head group. Each nanoparticle reached at the lipid head group within first 50 ns of simulation time and stayed there for some time. This indicated that there might be some kind of free energy barrier at the interface of lipid head group and water. As the AuNP reached the bilayer head group, the bilayer extruded slightly upward to interact with the AuNP. Once the bilayer head group and AuNP began to interact, the nanoparticles started penetrating the bilayer. The AuNP penetration was spontaneous after absorbing on the bilayer head group. The head groups of upper leaflet of the bilayer, which were just beneath the AuNPs, also moved along with the AuNPs towards the bilayer center. All AuNPs disrupted the bilayer packing and entered into the bilayer within 200 ns. Smaller AuNPs entered faster inside the bilayer interior because of their higher diffusivity as compared to bigger one. Li *et al*.^41^ reported similar phenomena in the simulation study of dipalmitoylphosphatidylcholine (DPPC) bilayer with 10 nm hydrophobic nanoparticle. The time taken by bare AuNPs (1–6 nm) to reach in the interior of the skin was in the order of ~ 80 ns^29^. It should be noted that the in previous simulations^29^ the surface of bare gold nanoparticles were highly hydrophobic and the mass of those gold nanoparticle was less as compared to coated one used here. Lin *et al*.^26^ reported the spontaneous penetration of 2.2 nm AuNP through mixture of DPPC and 1,2-dipalmitoyl-sn-glycero-3-phosphoglycerol (DPPG) bilayer. Huang *et al*.^17^ performed permeation experiments on mouse skin and showed that 5 nm AuNP penetrated rapidly through the epidermis of the mouse skin. Additionally, Sonavane *et al*.^16^ performed *in-vitro* permeation experiments of AuNPs of different sizes (~15, 102, 198 nm) through rat skin and showed that the permeation of 15 nm AuNP was higher than other bigger sized AuNPs. Another observation which is in line with our results is that the 15 nm AuNP could pass more rapidly from the outer layer to the deeper area of the skin as compared to bigger AuNPs. Labouta *et al*.^18^ performed permeation experiments of thiol coated 6 nm AuNP through human skin. It was shown that these AuNP penetrated into the SC and later migrated to deeper layers of the skin. Labouta *et al*.^19^ also investigated the human skin penetration of AuNPs with different surface modifications, sizes, vehicles, and concentrations. Non-polar (dodecanethiol coated ~6 nm, cetrimide-coated ~15 nm) and negatively charged (citrate coated ~15 nm) AuNPs were applied to the skin and incubated for 24 h. Neutral hydrophobic dodecanethiol and cetrimide coated AuNPs were able to penetrate to the deeper layer of skin but citrate coated AuNP were not detected in the deeper layer. Each neutral hydrophobic AuNPs disrupted the packing of head groups and lateral packing of bilayer chains and entered in the bilayer interior. Once the AuNPs reached in the interior of bilayer, they remained there for the entire duration of simulation and translocation of AuNPs towards outside (water region) was not observed. Ding *et al*.^42^ performed DPD simulation of DPPC bilayer in presence of ligand coated 3 nm hydrophobic nanoparticle and showed that nanoparticle took 12 μs for complete permeation from the top to bottom leaflet of the bilayer. In this study, we have observed incomplete permeation of AuNPs because of two reasons (i) simulations were run for 3 μs and (ii) the skin lipid bilayer was in gel phase as compared to liquid phase DPPC bilayer^42^.

The 2 nm AuNP did not induce much significant structural changes in the bilayer while bigger AuNPs (3–5 nm) changed the structure and packing, significantly. Some of the lipids from the bottom leaflet of the bilayer extended out and formed a hump-like structure to accommodate the AuNPs (Fig. 2b). The size of hump increased with the AuNP size. The AuNPs also induced undulation in the bilayer which increased with the AuNP size. The AuNP created vacancies in the bilayer interior but these vacancies were not able to transport water. The radial distribution profile of CHOL-AuNP (see supporting information, Fig. S1a) inferred that the created vacancies were mostly surrounded by the CHOL molecules. The peak position and intensity increased with the AuNP size. In contrast to our result, Gkeka *et al*.^43^ showed that the anionic nanoparticles depleted CHOL in the mixed bilayer of DPPC and CHOL. The polar head group of the DPPC bilayer was pulled by negatively charged ligands of nanoparticle^43^. It should be noted that in our case both the nanoparticle and lipids are uncharged and the nanoparticle is also hydrophobic in nature. The uncharged coated AuNPs attracted more non-polar CHOL molecules toward itself. Similar phenomena has also been observed in recent study^29^ of bare gold nanoparticle with skin lipids but the magnitude of peaks were more than the reported here.

**Figure 3:**
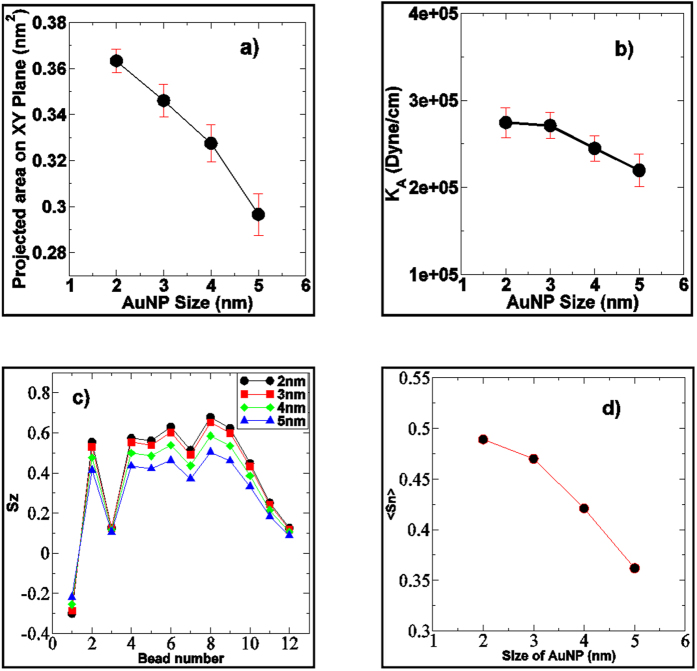
Result of effect of size of neutral hydrophobic AuNPs on skin bilayer properties. (**a**) Projected area on *XY* plane (**b**) area compressibility (**c**) order parameter Sz of CER chain sn1 and sn2 and (**d**) over all tail order parameter < *Sn > *in the presence of thiol coated AuNPs of size of 2–5 nm. The chain sn1 and sn2 are and corresponding bead numbers are shown in Fig. 1. For color code refer to web version of the article.

The bilayer packing was disturbed by each AuNPs, as shown in Fig. 2a,b. To quantify the curvature of the bilayer, Thake *et al*.^44^ proposed a protocol based on the radius of gyration of the polymer nanoparticle. We have employed a simplistic approach based on the projected area of the bilayer on *XY* plane. The details of the method has been given in the supporting information. The projected area on *XY* plane gives a qualitative picture of the bilayer curvature. The curvature or undulation in the bilayer will lead to less projected area as compared to normal bilayer (no undulation and curvature). The computed projected area, for each system, are shown in Fig. 3a. It is interesting to note that the area per lipid decreased with increase in the AuNPs size. This could be due to the undulation created by the AuNPs in the bilayer (Fig. 2b). As the extent of undulation increased with the AuNP size, the size of projected area decreased. Earlier, Ramalho *et al*.^45^ reported that a hydrophobic 3 nm nanoparticle decreased area per lipid of gel phase DPPC bilayer. The bilayer became softer with increase in AuNPs size as shown by the area compressibility in Fig. 3b. The order parameter and overall parameter are shown in Fig. 3c,d, respectively. The order parameter was calculated using an in house python script^29^. The shape of order parameter profile of each system was similar in nature. The order parameter decreased with increase in the AuNP size. The head group beads were in the anti-alignment with the bilayer normal while tails were aligned with bilayer normal z. The order parameter increased for sn1 beads while moving towards the center of the bilayer. The order parameter for a given bead and overall order parameter of chains decreased with the size of AuNPs, implying more disorder created by the bigger AuNPs in the bilayer.

### Effect of Surface Charge: Unconstrained Simulation

**Figure 4:**
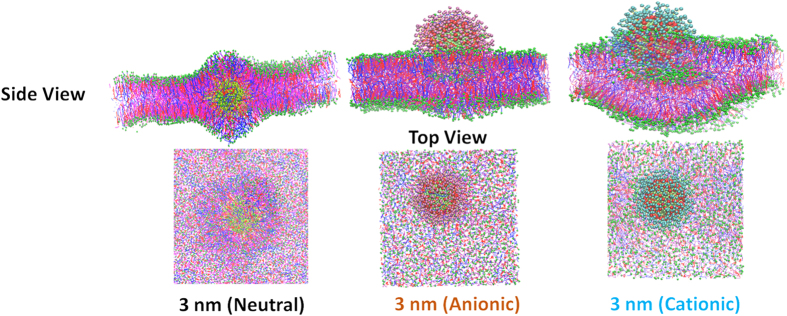
Result of interaction of neutral hydrophobic, cationic and anionic 3 nm AuNP with skin lipid bilayer. Snapshot of interaction of thiol coated neutral hydrophobic, anionic and cationic 3 nm AuNP with skin bilayer in the end of 3 μs unconstrained simulation run. The head groups of lipids (CER, CHOL and FFA), CER tail, CHOL aromatic rings, FFA tail, thiol chain and AuNP are shown in green, magenta, red, blue, yellow and dark green color respectively. The anionic and cationic AuNP are shown in VDW style and thiol chains of these AuNPs were represented by pink and cyan color respectively. Water molecules were removed for the purpose of clarity. Images/snapshots were created using the VMD software^59^.

Figure 4 shows the snapshot of the neutral hydrophobic and charged 3 nm AuNP with skin lipid bilayer after the 3 μs unconstrained simulation. The neutral hydrophobic AuNP penetrated inside the interior of the bilayer within the first 200 ns of simulation while both cationic and anionic AuNP were adsorbed near the head group and remained there for rest of the simulation time. To check the simulation time effect, the charged AuNP-bilayer systems were run for 6 μs but permeation of charged AuNP inside the bilayer was not observed (See supporting information, Fig. S2). Our results are aligned with earlier simulation studies of charged nanoparticle and AuNP with neutral cell membrane^26^,^28^,^46^,^47^. Lin *et al*.^26^ showed that the cationic and anionic dodecanethiol coated 2.2 nm AuNP adsorbed on the surface of neutral DPPC bilayer. They also showed that electrostatic interaction was crucial for the condition of AuNPs in the bilayer. The cationic AuNP was adsorbed on both neutral and negatively charged cell membrane surfaces while anionic AuNP was adsorbed only on the neutral bilayer^26^. Li and Gu showed that both positively and negatively charged nanoparticles (~6.8 nm) adsorbed on the head group of neutral DPPC bilayer^46^. It was reported that the electrostatic attraction improved the adhesion of a charged nanoparticle to the membrane. In addition, with the increase of electrostatic energy, a charged nanoparticle was wrapped by the membrane^46^. da Rocha *et al*.^47^ showed that charged AuNP adsorbed on the DPPC bilayer surface^47^. Simonelli *et al*.^28^ stated that anionic AuNP penetrated deeper in the bilayer. The AuNP−bilayer interaction was a three-step process: electrostatics driven adhesion to the membrane surface, hydrophobic contact and final embedding in the membrane core through anchoring of the charged ligands to both membrane leaflets^28^. In contrast, the penetration of charged AuNP in the bilayer interior is observed. Simonelli *et al*.^28^ used POPC bilayer which had highly charged head group as compared to neutral skin lipids used here. Experimentally, Labouta *et al*.^19^ showed that neutral hydrophobic dodecanethiol coated AuNPs (6 nm) were able to penetrate to the deeper layer of skin but citrate-coated AuNPs (negatively charged, 6 nm) were not detected in the deeper layer. Our findings are in line with these experimental results.

### Effect of Size: Constrained Simulation

**Figure 5:**
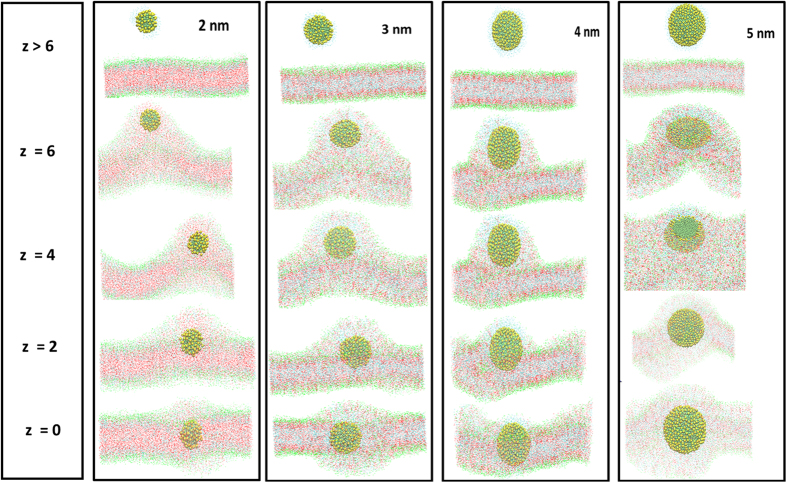
Result of interaction of constrained neutral hydrophobic AuNPs with skin lipids bilayer in constrained MD simulation. Final snapshot of thiol coated AuNPs of size of 2–5 nm bilayer (at different z position) in the end of 100 ns constrained simulation run. The head groups of lipids (CER, CHOL and FFA), CER tail, CHOL aromatic rings, FFA tail, thiol chain and AuNP are shown in green, magenta, red, blue, yellow and dark green color respectively. Water molecules were removed for the purpose of clarity. Images/snapshots were created using the VMD software^59^. Here z = 0 correspond to the bilayer centre.

Thermodynamic and transport properties of the AuNPs along the bilayer normal were calculated using several constrained MD simulations. Figure 5 shows the final snapshot of constrained simulation of bilayer in presence of thiol coated AuNPs of size of 2–5 nm bilayer constrained at different z position. The bilayer moved in the upward direction to interact with the AuNP when the distance between the distance of AuNP and the bilayer center was less than 6 nm. The bilayer completely engulfed the AuNP when it was constrained in the bilayer interior. This kind of phenomena was also shown by the Thake *et al*.^44^ when polymer nanoparticle of different size were simulated with DPPC bilayer. The projected area per lipid (on *XY* plane) is plotted along the bilayer normal (Fig. S3a). It is interesting to note that while moving from the bulk water to the interface, the projected area first remained constant (z > 6), then decreased and then increased slowly. The reason could be curvature induced by the AuNPs. The profile was found to be similar for each AuNPs but at a given z position, the projected area was found to be higher in smaller AuNP-bilayer system. The overall order parameter also followed the similar trend as plotted in Fig. S3b. (see supporting information)

**Figure 6:**
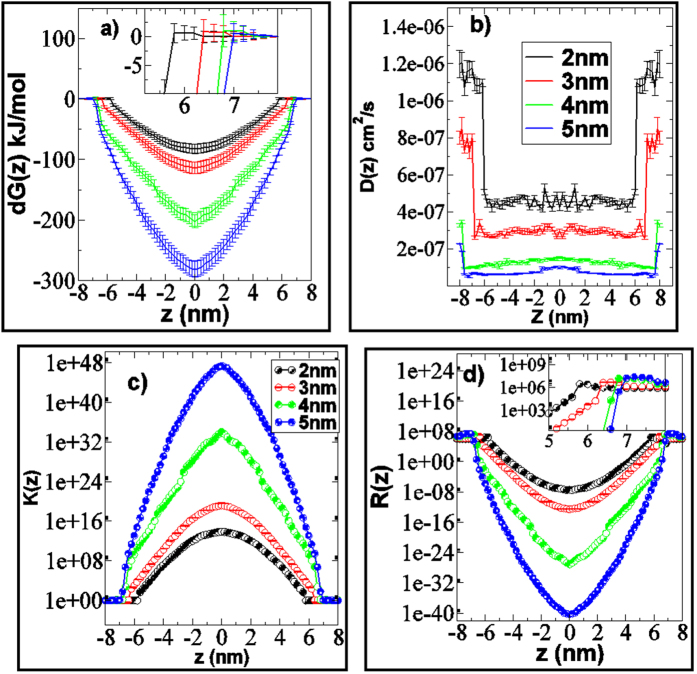
Results of thermodynamic and kinetic properties of permeation of neutral hydrophobic AuNPs through skin lipid bilayer. (**a**) Free energy of permeation (*dG*) (**b**) Diffusion coefficient *D(z)* (**c**) Partition coefficient *K(z)* and (**d**) Resistance of permeation *R(z)* of thiol coated neutral hydrophobic AuNPs of size of 2–5 nm along the bilayer normal (*z*), calculated from constrained CG MD simulation. Here *z* = 0 correspond to the bilayer centre. Bilayers were assumed to be symmetric and profile in one leaflet (upper) was replicated in another leaflet. For color code refer to web version of the article.

The free energy of permeation of AuNPs along the bilayer normal calculated from the CG constrained MD simulations, are plotted in Fig. 6a. The shape of permeation free energy profile of each AuNPs was similar in nature. The free energies were flat in the water phase and a very small barrier was observed near the head group for each AuNP (inset of the Fig. 6a). This explained why AuNPs remained at the head group of the lipids for most of the time before penetrating inside the bilayer in unconstrained simulation (Fig. 2a). Fiedler *et al*.^48^ reported similar small barrier near the interface of DPPC bilayer with hydrophobic carbon nanoparticle. Thake *et al*.^44^ also reported similar phenomena in case of hydrophobic polymer nanoparticle permeation through DPPC bilayer. Once the AuNP crossed the interface, the free energy decreased while moving towards the bilayer center. This confirmed that the bilayer interior is the favorable position for hydrophobic neutral AuNPs. The sharp negative gradient in the free energy near the head group led to quick permeation of the AuNP inside the bilayer interior as observed in unconstrained simulation (Fig. 2a). The difference of the free energy (magnitude) between the bulk water and the bilayer interior was maximum for the 5 nm AuNP and minimum for 2 nm AuNP. The bigger particle provides more hydrophobic surface area as compare to smaller one and the larger hydrophobic surface area is favorable for the hydrophobic lipid chains. Experimentally, Hong *et al*.^49^ shown the effect of position of tryptophan in a lipid bilayer by targeting the stability of the β-barrel membrane protein OmpA. It was shown that lipid solvation of tryptophan stabilized OmpA at all depths, but most stabilized when placed near the center of the bilayer^49^. The stability also gradually weakened as this residue was moved toward either membrane interface^49^. The large hydrophobic surface area were thought to be one of the possible reasons for these phenomena.

Our observations are in line with some of the recent findings on nanoparticle interaction with cell membrane and skin lipid bilayer as well. Lin *et al*.^50^ performed CG MD simulation of DPPC bilayer with three hydrophobic nanoparticles of different sizes ranging from 1.284 nm to 2.912 nm. It was reported that thermodynamics quantity such as free energy decreased with the increasing size of nanoparticles. No energy barriers were observed in the free energy profiles during the process of nanoparticle transport^50^. The free energy of permeation of 2.912 nm nanoparticle was ~−450 kJ/mol in the interior of the bilayer, while we have obtained ~−135 kJ/mol for 3 nm neutral hydrophobic AuNP. Ekkabut *et al*.^51^ carried out CG MD simulation of permeation of both single fullerene and fullerenes cluster through DPPC bilayer and showed that a very small free energy barrier existed near the headgroup (water-lipid interface) for hydrophobic fullerene particle. The permeation of both hydrophobic fullerene molecules and clusters was spontaneous as observed here in our simulation. The free energy in the bilayer interior was −100 kJ/mol for single fullerene molecule (~1.2 nm) while we have obtained ~−70 kJ/mol for 2 nm neutral hydrophobic AuNP. Thake *et al*.^44^ performed CG permeation simulation of four polyester nanoparticle of different size ranging from 1.3 nm to 3.7 nm through DPPC bilayer. They reported a very small barrier in the free energy profile near the headgroup of the lipid bilayer. We have also observed a very small barrier near the head group of the lipid membrane. The free energy of permeation of 2.7 nm nanoparticle was around ~ −1000 kJ/mol^44^ in the middle of the bilayer but we have obtained ~ −350 kJ/mol for 5 nm AuNP. It was also shown that the free energy of permeation was less for the 2.8 nm nanoparticle as compared to 3.7 nm. They have shown that the 3.7 nm nanoparticle was almost comparable to the bilayer thickness and had contact with the water from both side of the bilayer, which increased the free energy. In our simulations we observed that the AuNPs were completely engulfed by the bilayer and no contact with the water was found (Fig. 5). Van Lehn *et al*.^52^ reported the free energy of embedding the monolayer protected AuNPs of different sizes in the interior of the bilayer. The free energy minimum decreased with the AuNP diameter (1 nm to ~5 nm) 52. The magnitudes of the free energy minimum were lesser than similar sized neutral hydrophobic AuNP in this study. Fiedler *et al*.^48^ carried out constrained MD simulation of permeation of C60 (~1.25 nm) through DPPC bilayer and a very small barrier existed near the headgroup for permeation of hydrophobic C60 nanoparticle. The permeation free energy in the bilayer interior was around ~150 kJ/mol while in this study we have obtained −70 kJ/mol for 2 nm neutral hydrophobic AuNP. In above mentioned computational studies^44^,^48^,^50^,^51^,^52^, the free energy of permeation in the bilayer interior was almost two to three times lower than that of similar sized neutral hydrophobic AuNP used in this study. The reason could be that the DPPC bilayer used in earlier studies^44^,^48^,^50^,^51^,^52^ was at above the gel to liquid phase transition temperature while in our simulation the skin lipid bilayer was in gel phase. The free energy of permeation of water through gel phase pure ceramide skin layer^53^,^54^ (~ 47.6 kJ/mol) and liquid phase DPPC bilayer^55^ (~22.9–26 kJ/mol) has been reported earlier. Li *et al*.^56^ carried out DPD simulation of ligand coated nanoparticle through DPPC layer. The obtained free energy of permeation profile had similar as obtained in our simulation^56^. Gupta and Rai performed CGMD simulation of permeation of bare hydrophobic gold nanoparticle (1 nm-6 nm) through skin lipid bilayer and showed that free energy of permeation decreased with AuNP size^29^. The permeation of each AuNP was spontaneous and bigger AuNP took more time to reach the bilayer interior^29^. The experimental studies have shown that the 15 nm cetrimide coated AuNP penetrated to deeper skin layers due to their hydrophobic properties^16^,^17^,^19^. Experimental study by Labouta *et al*.^19^ suggested that the 6 nm thiol coated AuNP penetrated deeper layer of the skin. *In vitro* permeation studies of 15 nm AuNP on rat skin also showed that they were permeable^16^. Our simulations confirm that the neutral hydrophobic AuNPs have negative free energy of permeation inside the bilayer which lead to their permeation into deeper layers.

The diffusion coefficients (calculated using equation (2)) of each AuNP along the bilayer normal (z) are shown in Fig. 6b. The diffusion coefficients were found to be maximum in the bulk water and decreased in the interior of the bilayer. The shape of diffusion profiles was similar for each AuNP. Interestingly the diffusivity values were found to be almost same for a given size AuNP inside the bilayer interior. Fiedler *et al*.^48^ obtained relatively constant diffusion constant profiles for carbon nanoparticle in the interior of DPPC bilayer. The independency of diffusion to permeate size, in the bilayer interior, as compared to the water, was previously reported^57^, and relatively constant values along with the bilayer normal were previously reported for a nifedipine drug analog^58^. However, the diffusion constant values of the small molecular permeates changed with respect to z, and it has been correlated to membrane heterogeneity (differences in free volume)^54^,^55^,^57^. The diffusion values were found to be higher for smaller AuNP as compared to larger nanoparticles. At any given z position, the *D* value was found to be highest for the 2 nm AuNP and minimum for 5 nm AuNP. Lin *et al*.^50^ observed similar phenomena in the CG MD simulation of interaction of hydrophobic nanoparticles of size of 1.284, 2.098 and 2.912 nm with DPPC bilayer. The partition coefficients (calculated using equation (3)) of each AuNP along the bilayer normal (z) are shown in Fig. 6c. The *k(z)* profiles are complimentary to the free energy profiles for each AuNP. The bigger 5 nm AuNP had higher *k(z)* for any given z position as compared to other AuNPs. Fig. 6d shows the overall resistance of permeation (calculated using *R(z) = 1/k(z)D(z)*) of each AuNP along the bilayer normal z. The shape of *R(z)* profiles of each AuNP was similar to their respective free energy profile *dG(z)*. The *R(z)* profiles were similar to that observed in the permeation of hydrophobic carbon nanoparticle through DPPC bilayer^48^. The main resistance to permeation was in the water phase and near to the head group region. Negligible resistance was offered by the bilayer interior to each AuNP. The *R(z)* values increased near the headgroup as compared to bulk water for each AuNP that validated the observation (non-spontaneous permeation) noted in unconstrained simulation. (Fig. 2a)

### Effect of Surface Charge: Constrained Simulation

**Figure 7:**
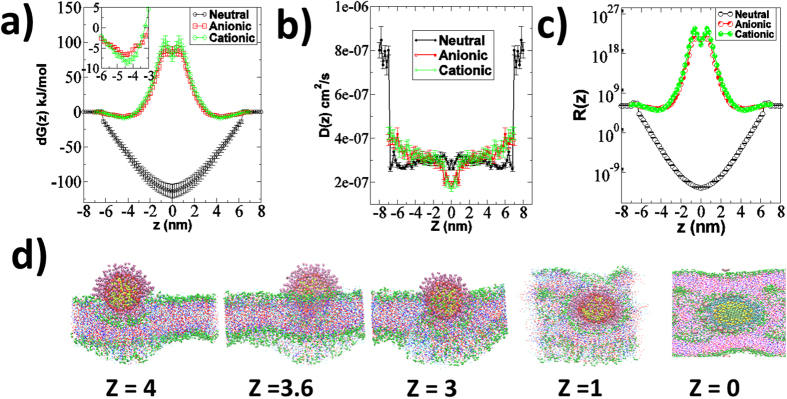
Results of thermodynamic and kinetic properties of permeation of neutral hydrophobic, cationic and anionic 3 nm AuNP through skin lipid bilayer. (**a**) Free energy of permeation *(dG)* (**b**) Diffusion coefficient *D(z)* (**c**) Resistance of permeation *R(z)* of thiol coated neutral hydrophobic, cationic and anionic 3 nm AuNP along the bilayer normal (*z*) calculated using constrained CG MD simulation. (**d**) Final snapshot of 1 μs unconstrained simulation of skin bilayer in the presence of 3 nm anionic AuNP at different z constrained position. The initial configuration for these simulation, was the final structure of the constrained simulation at given z position. Here z = 0 correspond to the bilayer centre. Bilayers were assumed to be symmetric and profile in one leaflet (upper) was replicated in another leaflet. Water molecules were removed for the purpose of clarity. Images/snapshots were created using the VMD software^59^. For color code refer to web version of the article.

Figure 7 shows the results of interaction of hydrophobic, cationic and anionic 3 nm AuNP in constrained simulation. The free energy of permeation of these AuNPs is potted in Fig 7a. The free energy profiles of both charged AuNPs were similar in shape. It is interesting to note that a very small barrier was observed near the head group for the neutral hydrophobic AuNP (also shown in the inset of Fig. 6a) while a minimum was observed near the headgroup for the charged AuNPs. It explained the adsorption and penetration of the charged and neutral hydrophobic AuNPs, respectively. Lin *et al*.^26^ performed CG MD simulation of DPPC bilayer with 2.2 nm AuNP of different surface charge. They reported that cationic and anionic nanoparticle had negative free energy near the headgroup of the DPPC bilayer and charged AuNPs adsorbed on the surface of the bilayer in unconstrained simulation^26^. The reported free energy of permeation of charged AuNPs were in the order of ~100 kJ/mol^26^ while we have obtained in the order of −10 kJ/mol, the possible reason could be DPPC bilayer temperature was maintained above the gel to liquid phase transition temperature while here, skin lipid bilayer was in gel phase. Li and Gu^46^ also showed that at higher charge density both cationic and anionic nanoparticle had positive free energy in the interior of the DPPC bilayer. Figure 7b,c shows the diffusion and resistance of permeation of each AuNP along the bilayer normal. The diffusion profiles were almost in similar in nature for each AuNP. The main resistance of permeation of neutral hydrophobic 3 nm AuNP was in water phase while for charged particles it was observed in the bilayer interior. The free energy of permeation of the charged AuNP was positive in the interior of the bilayer as shown in Fig 7a. The charged AuNPs adsorbed on the headgroup of the lipid bilayer in unconstrained simulation as shown in Fig 4. To check whether the lipid headgroup is a favorable position for charged AuNPs, additional unconstrained simulations (1 μs each) have been performed on final configuration obtained from the constrained simulation at different z position. The final snapshot of the systems have been shown in the Fig. 7d. It is interesting to note that when AuNP was constrained near the headgroup it remained there while in case when it was in the bilayer interior, it tried to disrupt the bilayer. These results confirmed that the charged AuNPs were adsorbed only on the headgroup while neutral hydrophobic AuNP penetrated inside the bilayer. Experimentally, Labouta *et al*.^19^ investigated the human skin penetration of AuNPs with different surface modifications, sizes, vehicles, and concentrations. Non-polar (dodecanethiol coated ~6 nm) and negatively charged (citrate coated ~15 nm) AuNPs were applied to the skin and incubated for 24 h^19^. Neutral hydrophobic dodecanethiol coated AuNPs were able to penetrate to the deeper layer of skin but citrate coated AuNP were not detected in the deeper layer^19^. In experiments, both neutral hydrophobic and charged AuNPs were dispersed in the water as done in our simulations. It has been shown earlier that the not only size of AuNPs but also the solvent and charge of AuNPs can affect skin penetration^19^.

### Permeability: Neutral Hydrophobic and Charged AuNP

The permeability of both neutral hydrophobic and charged AuNPs is listed in Table 2. For the neutral hydrophobic AuNPs the permeability decreased with the increased in the AuNP size. Our results are in agreement with available experimental studies. Sonavane *et al*.^16^ reported *in vitro* permeation of three different sized AuNPs (15, 102 and 198 nm) through rat skin. It was shown that the 15 nm AuNP was very much permeable compared to other two AuNPs. Huang *et al*.^17^ carried out experiments on rat skin and showed that 5 nm gold nanoparticles were able to penetrate deeper in the layer. Labouta *et al*.^18^ reported that the 6 nm AuNP showed greater extent of the penetration than 15 nm AuNP. The thermodynamics (free energy) and kinetics (diffusion) played important role in permeability of AuNPs through skin bilayer. The shape of the resistance of permeation was influenced by the free energy but the magnitude was controlled by the diffusion (Fig. 6d). In case of neutral hydrophobic AuNPs, the free energy of permeation was maximum for the 5 nm AuNP but the permeability was minimum. The free energy profile shows no barrier in the interior of the bilayer for neutral hydrophobic AuNPs so diffusion controlled the permeability. Similar phenomena has also been reported earlier for hydrophobic nanoparticle and hydrophobic molecules permeation through bilayer^29^,^54^. The free energy for each AuNP in the interior of the bilayer was negative (energetically favorable) but the diffusion coefficients decreased significantly with increasing size. Thus the combined effect of free energy and diffusion led to the higher permeability of small neutral hydrophobic AuNP as compared to bigger one. The charged AuNPs were almost impermeable as compared to same sized neutral hydrophobic AuNP. Experimentally, Labouta *et al*.^19^ showed that neutral hydrophobic dodecanethiol coated AuNPs (6 nm) were able to penetrate to the deeper layer of skin but citrate-coated AuNPs (negatively charged, 6 nm) were not detected in the deeper layer. Our findings are in line with these experimental findings.

## Conclusions

In this study, constrained and unconstrained CG MD simulations were performed to get the molecular level insight of interaction of thiol coated neutral hydrophobic (2, 3, 4 and 5 nm), cationic and anionic AuNPs (3 nm) with skin lipid membranes. The skin model membrane was modelled as an equimolar composition of CER, CHOL and FFA. Each neutral hydrophobic AuNPs penetrated deep in the bilayer interior while both cationic and anionic AuNPs did not get into the bilayer. The charged AuNPs adsorbed on the surface of the bilayer. Neutral hydrophobic AuNPs induced structural changes in the bilayer and created vacancies in the interior of the bilayer. The created undulation were quantified in terms of projected area per lipid (on *XY* plane), which decreased with increase in the size of neutral hydrophobic AuNP. Neutral hydrophobic AuNPs reduced the local and over all order parameter of the bilayers or in other words created more disruptions in the bilayer. The constrained simulation of neutral hydrophobic AuNPs also showed that the overall order parameter and projected area per lipid decreased along the bilayer normal on going from water to interface and increased from interface to bilayer center. The free energy of permeation was found to be maximum for 5 nm neutral hydrophobic AuNP while highest diffusivity was observed for 2 nm neutral hydrophobic AuNP at a given z position in the bilayer. No resistance of the permeation was observed in the bilayer interior for each neutral hydrophobic AuNP. The smaller neutral hydrophobic AuNP was more permeable as compared to bigger one. The charged AuNPs experienced main resistance to permeation in the bilayer interior. A free energy minimum was observed at the head group of bilayer for the both cationic and anionic AuNP, which did allow charged AuNPs to adsorb on the headgroup. The charged AuNPs were almost impermeable as compared to same sized neutral hydrophobic AuNP. Our simulations presented some interesting molecular level insight which could be used to design novel transdermal drug delivery systems as well as effective cosmetics. This study provides a huge scope where one can engineer the surface of AuNPs to create different types of structural changes in the bilayer for delivering the targeted drug/proteins.

## Additional Information

**How to cite this article:** Gupta, R. and Rai, B. Effect of Size and Surface Charge of Gold Nanoparticles on their Skin Permeability: A Molecular Dynamics Study. *Sci. Rep.* **7**, 45292; doi: 10.1038/srep45292 (2017).

**Publisher's note:** Springer Nature remains neutral with regard to jurisdictional claims in published maps and institutional affiliations.

## Supplementary Material

Supporting Information

## Figures and Tables

**Table 1:** Molar ratio used in the simulations and corresponding number of individual molecules.

System	AuNP size (nm)	Number of Dodecanethiol Molecule	CER	CHOL	FFA	Water
CG		—	832	800	832	20480
CG-AuNP	2	80	832	800	832	30680
	3	150	832	800	832	40960
	4	280	832	800	832	61440
	5	410	832	800	832	61440

UA, CG and CG-AuNP stands for united atom, coarse grained and coarse-grained with AuNP bilayer system.

**Table 2:** Calculated permeability (*P*) of each AuNP through skin lipid bilayer.

System	AuNP size (nm)	Permeability (*P*)* (cm/s)
CG-AuNP	2	0.916 ± 0.124
	3	0.595 ± 0.108
	4	0.184 ± 0.056
	5	0.144 ± 0.062
	3 (Anionic)	(1.121 ± 0.112) × 10^−15^
	3 (Cationic)	(6.076 ± 0.341) × 10^−16^

^*^Equation (1)
