# Supplementary material for: Effect of Size and Surface Charge of Gold Nanoparticles on their Skin Permeability: A Molecular Dynamics Study
Source: Sci Rep. 2017 Mar 28;7:45292. doi: 10.1038/srep45292 (PMC5368607; doi:10.1038/srep45292)
Supplement: Supporting Information [file srep45292-s1.pdf]

Supporting Information

Effect of Size and Surface Charge of Gold Nanoparticles on their Skin Permeability: A Molecular Dynamics Study.

Rakesh Gupta and Beena Rai*

Physical Science Research Area, TCS Research

Tata Research Development and Design Centre, Tata Consultancy Services,

54B, Hadapsar Industrial Estate, Pune – 411013, INDIA

*Corresponding author: beena.rai@tcs.com

Fax: 91-20-66086399

Tel: 91-20-66086203

Projected area on XY plane

In a molecular dynamics simulation of lipid bilayer, which has normal along the z direction, the projected area per lipid (APL) on xy plane can be calculated using the following equation:

$$A = 2\frac{Lx\,Ly}{Nlipid} \qquad \text{(S1)}$$

Where L_x , L_y is the box length in X and Y direction, respectively and N_{lipid} is total number of lipids in the bilayer.

Order parameter

The second rank order parameter for the bilayer, which has normal in z direction, could be defined as:

$$Sz = \frac{1}{2}(3cos^2\theta - 1) \qquad \text{(S2)}$$

where θ is the angle between the bond and the bilayer normal. Sz = 1 means perfect alignment with the bilayer normal, Sz = −0.5 anti-alignment, and Sz = 0 random orientation of the lipid chains.

Overall order parameter along the bilayer axis Z

The overall order parameter was calculated using the following relationship:

$$S = \frac{\sum_{i=1}^{n} Sz(i)}{n} \qquad \text{(S3)}$$

Where n is number of beads in the ceramide molecules and Sz is order parameter for i^{th} bead of ceramide chain as shown in the Figure 1.

(a)

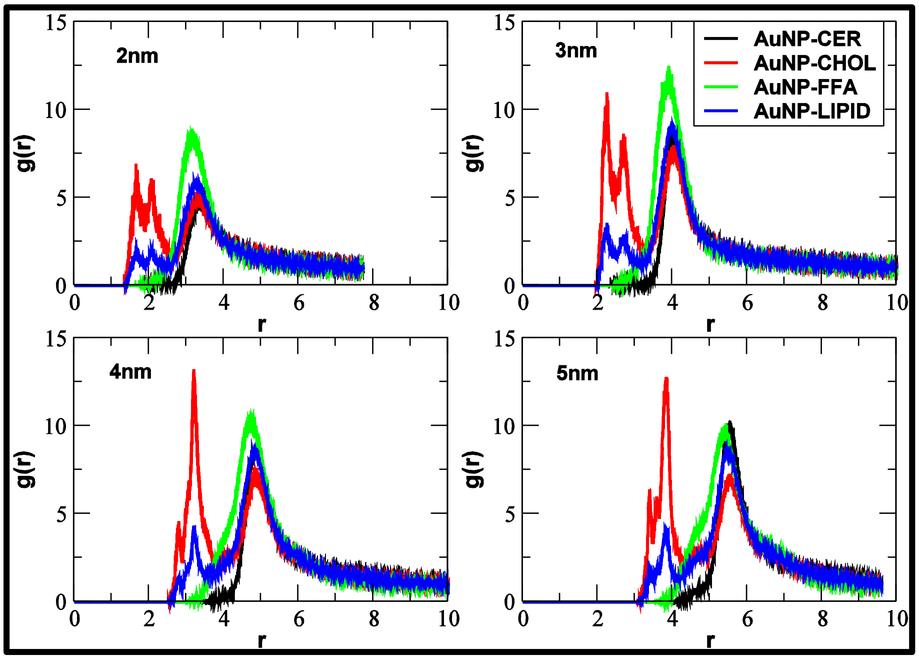

(b)

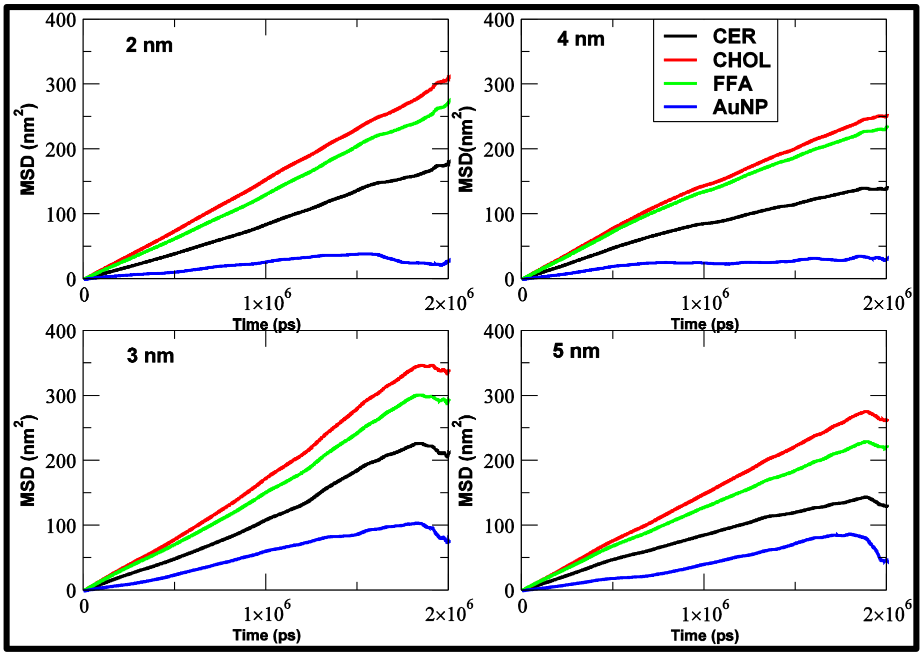

Supplementary Figure S1. Result of interaction of neutral hydrophobic AuNP with skin lipids: (a) Radial Distribution function *g(r)* of CER, CHOL, FFA and LIPID with respect to thiol coated AuNPs (b) mean square displacement (MSD) of CER, CHOL, FFA and AuNPs calculated in 3 μs unconstrained simulation. LIPID stands for combined CER, CHOL and FFA.

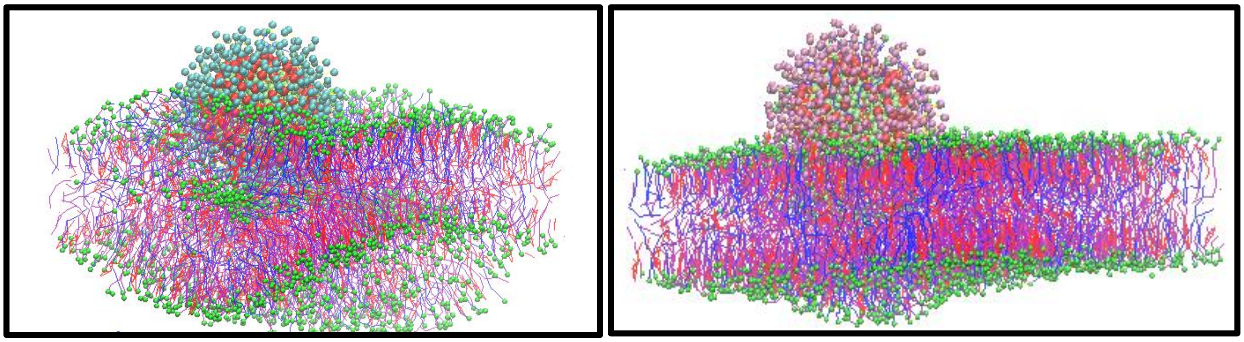

Supplementary Figure S2. Result of interaction of charged cationic and anionic 3nm AuNP with skin lipids: Snapshot of interaction of thiol coated anionic and cationic 3nm AuNP with bilayer in the end of 6 µs unconstrained simulation run. The head groups of lipids (CER, CHOL and FFA), CER tail, CHOL aromatic rings, FFA tail, thiol chain and AuNP are shown in green, magenta, red, blue, yellow and dark green color respectively. The anionic and cationic AuNP were sown in VDW style of VMD software and thiol chains of these AuNPs were represented by pink and cyan color respectively. Water molecules were removed for the purpose of clarity. Images/snapshots were created using the VMD software.[1]

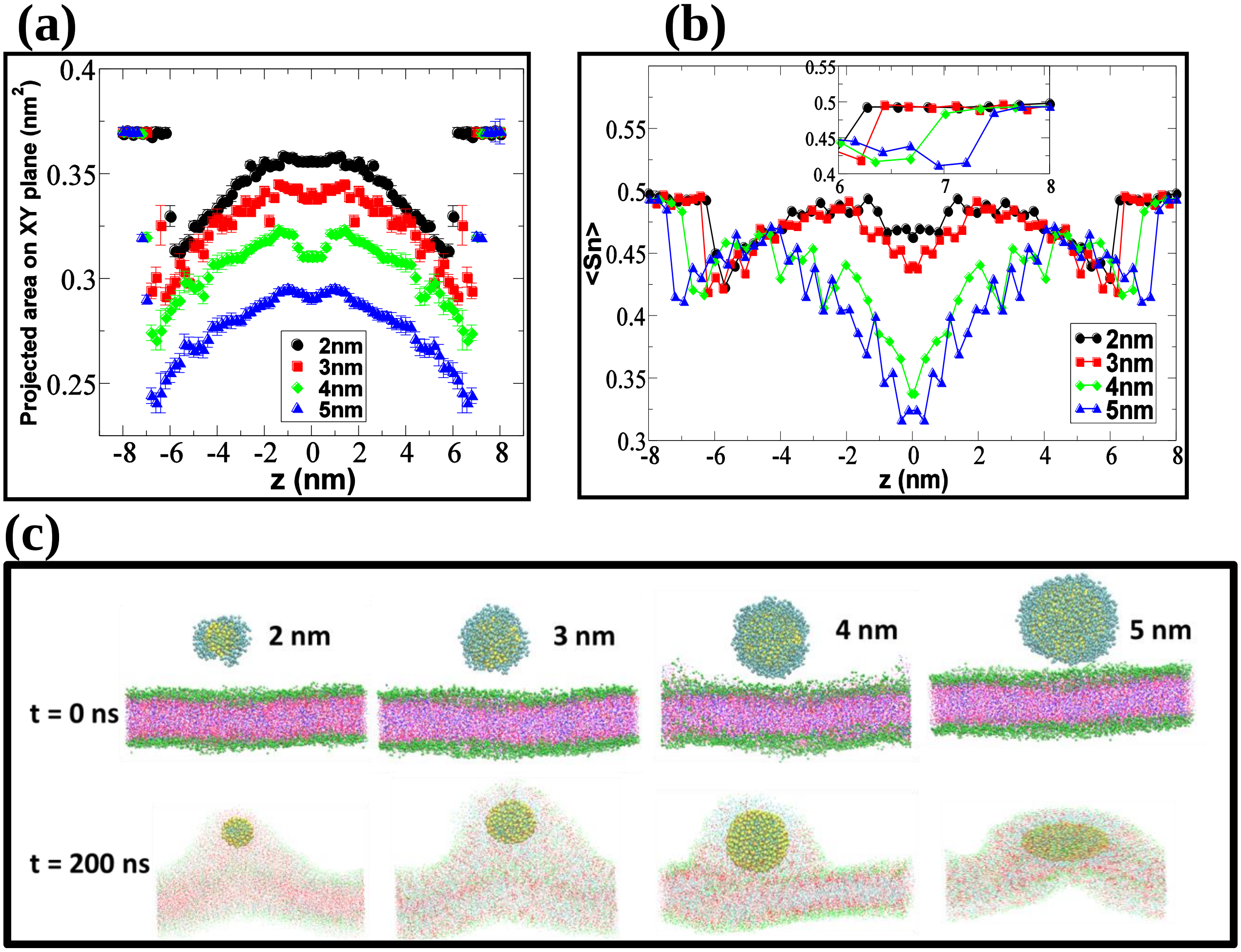

Supplementary Figure S3. Result of interaction of neutral hydrophobic AuNPs with skin lipids in constrained MD simulation: (a) The projected are on *XY* plane, (b) over all order parameter <*Sn*> of ceramide chains (c) Initial and final snapshot of thiol coated AuNPs of size of 2-5nm bilayer (at z=6) in constrained simulation run. The head groups of lipids (CER, CHOL and FFA), CER tail, CHOL aromatic rings, FFA tail, thiol chain and AuNP are shown in green, magenta, red, blue, yellow and dark green color respectively. Water molecules were removed for the clarity purpose. Images/snapshots were created using the VMD software.[1] Here z=0 correspond to the bilayer centre. Bilayers were assumed to be symmetric and profile in one leaflet (upper) was replicated in another leaflet. Chain sn1 and sn2 are shown in the Fig.1. For color code refer to web version of the article.

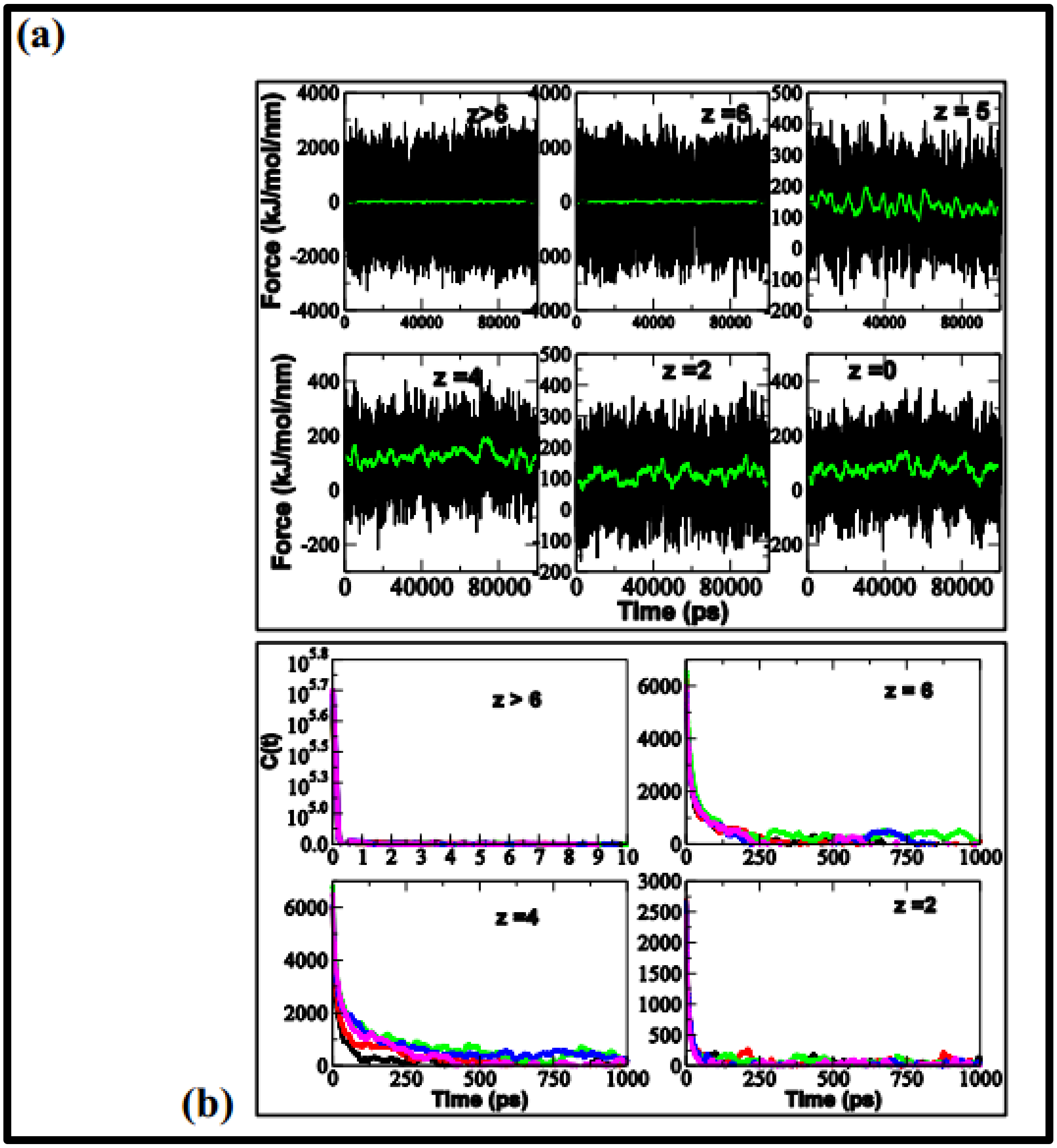

Supplementary Figure S4. Calculation of averaged constrained force, free energy of permeation and diffusion coefficient in constrained simulation: (a) Evolution of constrained force F_z (b) Autocorrelation function of constrained force F_z on each neutral hydrophobic 2nm AuNP at different z position in final 100 ns constrained simulation run. The green line shows the running average of the force. The running average of force calculated over 100 ps time interval.

The calculation of average force and force autocorrelation has been shown Fig S4. The green lines in Fig. S4a shows the running average of force at different z position. The averaged force at different z position, was integrated over the bilayer length using equation (5) to obtain the free energy of permeation. The non-normalized force autocorrelation ($<\triangle F(z,t) \triangle F(z,0)>$)

functions, calculated using g_analyze function of GROMACS[2-4] , are plotted in Fig. S4b. The autocorrelation decays almost exponentially with time. The autocorrelation function was further integrated and used for the diffusivity calculation by using equation (2).

References

1. Humphrey, W., Dalke, A. and Schulten, K. VMD - Visual Molecular Dynamics. *J. Molec. Graphics* ***14.1***, 33-38 (1996).
2. Hess, B., Kutzner, C., van der Spoel, D. & Lindahl, E. GROMACS 4: Algorithms for Highly Efficient, Load-Balanced, and Scalable Molecular Simulation. *J. Chem. Theory Comput.* **4,** 435-447 (2008).
3. Kutzner, C. et al. Speeding up parallel GROMACS on high-latency networks. *J. Comput. Chem.* **28,** 2075-2084 (2007).
4. Pronk, S. et al. GROMACS 4.5: a high-throughput and highly parallel open source molecular simulation toolkit. *Bioinformatics* **29,** 845-854 (2013).
